# Advancements in artificial intelligence for atopic dermatitis: diagnosis, treatment, and patient management

**DOI:** 10.1080/07853890.2025.2484665

**Published:** 2025-04-08

**Authors:** Fang Cao, Yujie Yang, Cui Guo, Hui Zhang, Qianying Yu, Jing Guo

**Affiliations:** ^a^ Chengdu University of Traditional Chinese Medicine, Chengdu, China; ^b^ Sinopharm Chongqing Southwest Aluminum Hospital, Beijing, China; ^c^ Hospital of Chengdu University of Traditional Chinese Medicine, Chengdu, China

**Keywords:** Atopic dermatitis, artificial intelligence, deep learning, skin disease, interdisciplinary

## Abstract

Atopic dermatitis (AD) is a common and complex skin disease that significantly affects the quality of life of patients. The latest advances in artificial intelligence (AI) technology have introduced new methods for diagnosing, treating, and managing AD. AI has various innovative applications in the diagnosis and treatment of atopic dermatitis, with particular emphasis on its significant benefits in medical diagnosis, treatment monitoring, and patient care. AI algorithms, especially those that use deep learning techniques, demonstrate strong performance in recognizing skin images and effectively distinguishing different types of skin lesions, including common AD manifestations. In addition, artificial intelligence has also shown promise in creating personalized treatment plans, simplifying drug development processes, and managing clinical trials. Despite challenges in data privacy and model transparency, the potential of artificial intelligence in advancing AD care is enormous, bringing the future to precision medicine and improving patient outcomes. This manuscript provides a comprehensive review of the application of AI in the process of AD disease for the first time, aiming to play a key role in the advancement of AI in skin health care and further enhance the clinical diagnosis and treatment of AD.

## Introduction

Atopic dermatitis (AD) is a chronic and recurrent inflammatory skin condition primarily characterized by severe itching and dryness. It involves epidermal barrier dysfunction and immune response disorders exacerbated by environmental factors [1]. The onset of AD is complex, involving genetic predisposition, environmental triggers, immune system irregularities, and defects in the skin’s epidermal barrier. This intricacy complicates the diagnosis and management of AD, presenting a significant clinical challenge [2]. Epidemiological data collected in 2022 indicate that approximately 10–20% of children and 2–10% of adults worldwide are affected by AD, with prevalence varying by region and ethnicity, and higher rates observed in developed countries [3]. Beyond its effects on the skin, AD significantly impacts the quality of life, causing sleep disturbances, emotional instability, and psychosocial challenges. Key clinical concerns related to AD include challenges in accurate diagnosis, therapeutic management, and long-term care. The disease’s diverse clinical manifestations can be mistaken for other conditions, such as psoriasis and contact dermatitis [4]. Diagnosis typically involves a detailed medical history, physical examination, and sometimes additional tests, further complicating the diagnostic process. AD is a chronic condition requiring prolonged treatment and regular adjustments to daily routines, diet, and skincare practices to control symptoms and prevent relapse effectively [5].

Currently, the diagnosis of AD primarily relies on clinicians’ experience and traditional methods such as skin biopsy [6]. These methods are time-consuming, and their accuracy can vary depending on the clinician, leading to imprecise and inconsistent diagnoses. Conventional diagnostic procedures are ineffective in distinguishing between skin lesions, which can delay diagnosis and classification, potentially resulting in misdiagnosis or missed diagnoses. Treatment options for AD include topical drugs such as glucocorticoids and calcineurin inhibitors, systemic treatments such as immunosuppressants and phototherapy, and biological agents [7]. Although these treatments can offer some symptom relief, their effectiveness is frequently limited and accompanied by side effects [7,8]. Patients with moderate to severe AD face challenges in finding effective long-term treatment with the current options. Prolonged drug use can lead to drug resistance and other side effects, further limiting treatment options. Moreover, managing AD is complicated due to its chronic nature, requiring adjustments to lifestyle, diet, and skincare routines. Most patients lack sufficient medical knowledge and expertise, resulting in suboptimal disease management, frequent relapses, and a significant reduction in quality of life [9,10]. Therefore, increasing patients’ self-management abilities and enhancing the quality of long-term care are crucial priorities.

Doctors and researchers have explored effective ways to diagnose and treat AD. Artificial intelligence (AI) technology has recently made significant advances, opening up more possibilities for the diagnosis and treatment of atopic dermatitis [11]. By leveraging deep learning and extensive data analysis, AI technology can offer more precise diagnostic capabilities. Moreover, AI technology provides advantages in personalized treatment and immediate patient care. AI has expanded into various domains, including medical image recognition, genomic analysis, drug discovery, and continuous patient monitoring [12,13]. In dermatology, AI algorithms have been applied to analyze and classify skin lesions with a diagnostic accuracy comparable to dermatologists. Furthermore, AI enables comprehensive analysis of extensive clinical data, helping to define diseases, predict their occurrence, and recommend tailored treatment plans for individual patients [14,15].

This manuscript presents the application of AI technology in diagnosing, treating, and managing AD. Moreover, it analyzes the advantages and disadvantages of these applications in clinical practice and considers the potential future advancements of AI in skincare.

## Application of AI in AD diagnosis

### Application of AI algorithms in skin image analysis

AI-based skin image recognition plays a crucial role in diagnosing AD. Previous findings indicate the effectiveness of AI algorithms, particularly deep-learning technologies, in analyzing skin images with remarkable accuracy. By training AI using extensive datasets of skin lesion images, these algorithms can accurately identify different types of skin lesions, including typical AD presentations [16]. Complex neural network architectures such as convolutional neural networks (CNNs) are used to analyze image features and patterns. In a study by Kim, hyperspectral imaging (HSI) was integrated with AI to enhance the diagnosis and classification of AD. They adopted parameter-based transfer learning, where the AI model was trained on datasets comprising psoriasis, eczema, and AD features. The study demonstrated that integrating the ResNet-50 model with hyperspectral imaging achieved the highest classification accuracy of 83% in diagnosing the severity of AD [17]. These observations indicate that integrating hyperspectral imaging with deep learning models significantly improves the accuracy of AD classification.

AI technology has significantly advanced lesion detection and classification, particularly in AD. Trained AI models effectively identify AD features from various skin images based on lesion color, shape, and texture. Maulana’s study collected images from 250 patients and evaluated five deep learning models, namely ResNet-50, VGGNet-19, MobileNetV3, MnasNet, and EfficientNetB0, for the automatic assessment of atopic dermatitis severity. The performance of these models was measured using metrics such as accuracy, precision, sensitivity, specificity, and F1 score. Among these, ResNet-50 demonstrated the best performance, achieving an accuracy of 89.80%, a precision of 90.00%, a sensitivity of 89.80%, a specificity of 96.70%, and an F1 score of 89.95% [18]. This shows that ResNet-50 has strong ability to identify complex patterns and is suitable for skin lesion image analysis. These findings demonstrate the effectiveness of deep learning models in diagnosing AD and support further advancements in this field. Also, these models hold promise in aiding dermatologists in accurately determining the severity of AD and ensuring patients receive proper care [19]. Furthermore, Medela conducted a pilot study using deep learning to score AD based on 604 skin images, achieving a diagnostic accuracy of 84.6% [20]. This study highlighted the role of deep learning in dermatology image classification and its potential to reduce bias and expand unbiased analysis in dermatology images. Moreover, Sojeong Park et al. developed a CNN model, The original 3D RSOM image is processed through data augmentation (Data Augmentation), including rotation and flipping operations, and then randomly crops a fixed-size area to generate more training samples and increase the generalization ability of the model. In addition, the researchers extracted four features: total blood volume (TBV), epidermal thickness (ET), low- and high-frequency signal ratio (LHFR), and transepidermal water loss (TEWL) to differentiate between healthy and eczema (AD) conditions, as well as mild AD and moderate to severe AD [21]. Using the 3D photoacoustic microscopy images of patients with AD, the CNN model achieved a high accuracy of 97% in distinguishing healthy individuals from patients with AD. Moreover, the random forest model demonstrated 65% accuracy in classifying mild and moderate AD cases compared to severe cases [21]. This research demonstrated the promise of photoacoustic microscopy in AD diagnosis and highlighted the application of various ML methods in diagnosing skin diseases.

In addition to deep learning methods, traditional machine learning algorithms also demonstrate unique value in the analysis of skin images related to atopic dermatitis (AD). Support vector machines (SVM) exhibit exceptional performance in diagnosing AD skin images, particularly due to their effectiveness with small sample datasets and their capability to manage nonlinear classification challenges. The SVM classifier, when based on texture features, shows high specificity and sensitivity in differentiating AD from other inflammatory skin lesions. Meanwhile, artificial neural networks (ANN) effectively capture the complex characteristics of skin lesions through their multi-layer perceptron architecture. Furthermore, ensemble learning methods enhance the accuracy and stability of AD diagnosis by integrating the strengths of multiple classifiers. The combined application of these traditional machine learning techniques alongside deep learning technologies offers a more comprehensive solution for the intelligent diagnosis of AD.

The application of AI for image analysis provides several advantages over traditional methods, primarily by improving diagnostic efficiency and minimizing the impact of human factors. Conventional diagnostic approaches depend heavily on the training and experience of individual doctors, which can vary and lead to inconsistencies [22]. In contrast, AI ensures uniformity in image analysis procedures, enhancing the efficiency and accuracy of clinical diagnosis.

### Application in other diagnostic tools

#### Genetic and biomarker analysis

Besides skin image recognition, AI holds promise in analyzing gene expression data to identify biomarkers associated with the disease and gain insights into its molecular characteristics. This capability lays a foundation for developing personalized therapies tailored to individual patients.

Dev et al. used gene expression data to explore skin barrier function and immune response in patients with AD. They employed an ML-assisted handheld confocal Raman microspectroscopy (CRM) system to analyze gene expression profiles in these patients comprehensively. The results showed the PLS-DA model achieved an accuracy of 92% in distinguishing patients from healthy individuals, with a sensitivity and specificity of 94% and 85% respectively [23]. This approach enhances the diagnostic performance for AD by identifying essential skin chemical components, such as proteins and lipids, in the skin surface layer.

Furthermore, faced with challenges in AD diagnosis, such as variable morphology, irregular distribution, and lack of objective and effective markers, researchers have proposed an innovative method. This method involves combining transcriptomic data of intestinal epithelial colon cells with intestinal microbiome data to develop a machine learning classifier for accurate automatic diagnosis of AD. The classifier, tested on 161 samples including 88 AD patients and 73 healthy controls, exhibited strong performance with an average F1 score of 0.84. A high F1 score indicates the model’s accuracy in distinguishing AD patients from non-patients, making it a crucial indicator for evaluating classifier performance [24]. The study not only showcases the potential of machine learning in diagnosing complex diseases but also suggests the possibility of identifying new biomarkers for early detection and personalized treatment of AD.

Through its integrated learning characteristics, the random forest (RF) algorithm effectively processes high-dimensional gene expression data, facilitating the screening and classification of genes associated with atopic dermatitis (AD). Research indicates that the RF model successfully identifies multiple key genes involved in the pathogenesis of AD when analyzing gene expression datasets, such as GSE120721. The Least Absolute Shrinkage and Selection Operator (LASSO), a powerful feature selection method, plays a crucial role in identifying key biomarkers related to AD. By combining LASSO with Support Vector Machine Recursive Feature Elimination (SVM-RFE), researchers have successfully identified a series of biomarkers closely associated with the onset and progression of AD, including CCR5 and its related transcription factors NR3C2 and YY1 [25]. These findings not only enhance the accuracy of AD diagnosis but also provide new molecular targets for targeted therapy. Furthermore, these algorithms demonstrate excellent performance in analyzing immune cell infiltration characteristics and cytokine networks, thereby aiding researchers in better understanding the immunopathological mechanisms underlying AD [26].

#### Medical record data analysis

Using AI to analyze medical history data is an important and expanding area of research. By applying natural language processing (NLP) technology, AI can extract crucial information from electronic health records (EHRs), providing physicians with a comprehensive overview of a patient’s condition and treatment history [27]. A study based on a large language model found that AI tools outperformed general practitioners’ responses in terms of quality and empathy in answering 99 common questions about atopic dermatitis patients provided by 11 international dermatologists [28]. Gustafson conducted a classification study using ML models and EHRs to predict adult patients with AD accurately. The study used Ontario’s health database to identify patients with dermatitis through keyword searches and diagnostic coding. Electronic medical records were manually audited to verify the accuracy of algorithmic identification, and multiple machine learning (ML) algorithms were used for text classification. Different methods for identifying AD in routine health data in Ontario were evaluated, highlighting the potential for using ML to improve diagnostic algorithms to more accurately identify AD cases [29]. By processing extensive medical history data using these ML algorithms, AI systems can effectively identify potential risk factors and trends associated with AD, aiding in accurate diagnosis.

#### Application of multimodal AI models

Multimodal artificial intelligence models are enhancing the accuracy and efficiency of diseases diagnosis by integrating various data sources, including image recognition, text analysis, and biomarker detection [30]. These models can analyze visual information from patient skin images and combine it with symptom descriptions, patient self-reported data, and biomarkers to enhance diagnostic reliability. By leveraging deep learning algorithms, multi-modal models can recognize and understand the intricate patterns of skin diseases, offering doctors more precise diagnostic assistance in the initial phases [31]. In a study, researchers created a software named MedIA (Medical Data Integration Assistant) capable of exploring and extracting specific data subsets for analysis. The MedIA system facilitates data management through the integration and visualization of AD patients data and information, assisting data scientists in accessing necessary datasets [32]. This approach facilitates efficient data management and subset selection, enabling researchers to identify the underlying pathological mechanisms of AD with greater ease and accuracy. Another pioneering example involved combining multiphoton tomography with CNN technology, The researchers collected 3,663 MPT images containing morphological and metabolic data from both AD patients and healthy volunteers. These images were used to train a CNN model capable of automatically identifying living cells and diagnosing AD. The algorithm achieved a 97.0% accuracy in detecting live cell images, with a sensitivity of 0.966 and a specificity of 0.977 [33]. Additionally, the study improved the interpretability of the algorithm by employing deep Taylor decomposition techniques, which highlighted important features in images for a specific classification using heat maps.

Compared to traditional single-modal methods, multimodal models demonstrate advantages on multiple levels. Unimodal methods are often constrained to a single data source, which may result in the omission or bias of diagnostic information. In contrast, the multimodal approach can more accurately identify features of Alzheimer’s Disease (AD) and mitigate the impact of false positives and false negatives by comprehensively analyzing data from multiple sources. In diagnosing AD, multimodal AI can integrate skin images and clinical symptoms, as well as genomic and microbiome data, to predict disease progression and develop personalized treatment plans [34]. This integrated approach significantly enhances clinical utility and accuracy. Although multimodal AI models exhibit considerable potential, they encounter challenges in practical applications, such as the complexity of data fusion, the necessity for cross-institutional data standardization, and the limited availability of data for model training. Addressing these issues requires strengthening interdisciplinary collaboration and developing more efficient algorithms, thereby further advancing the application of multimodal AI models in clinical diagnosis and treatment.

### Clinical advantages of AI-assisted diagnosis

Using AI in disease diagnosis offers significant benefits by improving the speed and reliability of diagnostic outcomes. Unlike conventional diagnostic approaches, AI systems efficiently process data and generate diagnostic results promptly, reducing assessment times [35,36]. This process optimizes diagnostic speed and improves the medical experience by minimizing patient waiting times. Several successful applications of AI in the diagnosis of AD are available. For example, A study from Spain demonstrated that the integration of AI and machine learning can effectively reduce the time required for SCORAD, a common diagnostic tool for atopic dermatitis, to detect and diagnose the condition while maintaining accuracy [20]. Additionally, research has shown success in utilizing AI and hyperspectral imaging (HSI) for diagnosing atopic dermatitis. This technology has significantly enhanced the diagnostic process by leveraging comprehensive data sets and advanced multi-frame algorithms. The model exhibited high sensitivity (90.72%), specificity (96.76%), and prediction accuracy in identifying atopic dermatitis, psoriasis, and other skin lesions [37]. These algorithms effectively differentiated AD from other skin disorders with high accuracy, comparable to or slightly exceeding standard dermatology practices in some cases. AI systems have demonstrated clinical effectiveness, particularly in targeted clinical scenarios and their implementation in real-world settings. Such systems contribute to increasing the accuracy of diagnostic systems and reducing the subjectivity in the process. Furthermore, research and clinical trials have shown that using AI in diagnosis significantly reduces misdiagnosis rates and enhances the credibility of clinical diagnostic systems [38]. At the same time, AI-based assessment systems offer superior objectivity and precision in evaluating AD severity compared to traditional human assessment, as they eliminate subjective bias and provide consistent, quantifiable measurements across different evaluations. This standardized approach is particularly valuable for tracking disease progression and treatment efficacy over time. Analyze from a long-term perspective, the potential for collaboration between medical practitioners and AI systems is substantial, paving the way for more effective healthcare delivery and improved patient outcomes.

Artificial intelligence systems have shown remarkable advantages in diagnostic accuracy and efficiency, with encouraging results such as ResNet-50 achieving 83% accuracy in disease severity classification and 3D optoacoustic microscopy combined with CNN reaching an impressive 97% accuracy. However, these technological advances face several key challenges: the reliability of AI diagnosis heavily depends on large-scale, high-quality datasets, which are often difficult to obtain due to privacy concerns and lack of standardization across institutions. Moreover, while studies show promising accuracy rates, most current research involves relatively small sample sizes and lacks extensive real-world validation against experienced dermatologists. A particularly promising direction is the multimodal AI approach, which combines image analysis, genetic data, and clinical records for more comprehensive diagnosis. However, implementing such systems requires significant infrastructure investment and careful integration into existing clinical workflows. Looking ahead, we believe the key to successful AI implementation in AD diagnosis lies in viewing it as a tool to enhance, rather than replace, clinical expertise. Future research should focus on larger multicenter studies, standardized data protocols, and clear guidelines for clinical integration while maintaining the crucial role of physician judgment in patient care.

**Figure 1.**
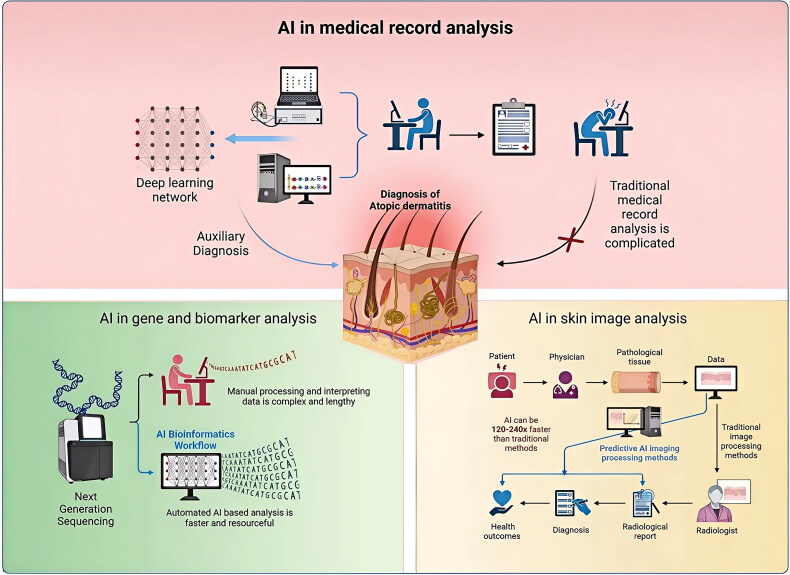
The role of artificial intelligence in diagnosis of atopic dermatitis.

By integrating AI into medical practice, doctors can harness AI’s robust data analytical capabilities alongside their personal experience and expertise in the medical field to enhance healthcare services. Integrating technology into the human-machine practice model is expected to be a prominent trend in future medical advancements, holding promise for improving early diagnosis and treatment of AD and other skin conditions (Figure 1 and Table 1).

**Table 1.** Summary of key studies on AI applications in atopic dermatitis.

Author & year	Study focus	AI method/model	Results/outcomes
Zarca et al. 2018	Tele-expertise for skin lesion diagnosis in prison setting	Al-based teledermatology system	Increased treatment plan completion rates
Guimarães et al. 2020	AD diagnosis using multiphoton tomography	CNN with deep Taylor decomposition	97.0% accuracy, 0.966 sensitivity, 0.977 specificity
Yamamoto-Hanada et al. 2020	Plasma cytokine/chemokine analysis	Machine learning analysis	Identified CCL17 and CCL27 associated with AD
Bang et al. 2021	AD severity scoring	Deep neural network	High accuracy comparable to dermatologist assessment
Park et al. 2021	3D optoacoustic mesoscopy analysis	CNN model	97% accuracy in AD detection, 65% for severity classification
Dev et al. 2022	Biomarker identification using Raman spectroscopy	ML-assisted CRM	92% accuracy, 94% sensitivity, 85% specificity
Jiang et al. 2022	Combined transcriptome and microbiota analysis	Supervised machine learning	F1 score of 0.84 across 161 samples
Li et al. 2022	AD prediction using bSRWPSO-FKNN	Boosted PSO with fuzzy KNN	Identified key biomarkers (LY, cat dander, milk, IgE)
Medela et al. 2022	Automatic SCORAD scoring	Deep learning	84.6% diagnostic accuracy
Wu et al. 2022	Dupilumab response prediction	Machine learning analysis	Identified 35% non-responders and predictive factors
Wang et al. 2022	Drug development (CMA analysis)	Network analysis-deep learning	Identified CMA as TNFα/IL-4 inhibitor for AD
Kern et al. 2023	Digital self-help intervention	AI-based CBT system	Effect size (Cohen’s *d* = 0.84) at 3-month follow-up
Maulana et al. 2023	AD severity evaluation	Multiple deep learning models	ResNet-50: 89.80% accuracy, 90.00% precision
Yamanaka et al. 2023	Drug development using DRAGONET	Deep learning for molecule generation	Generated new therapeutic molecular structures
Huang et al. 2024	Skin lesion identification	HSI with Al	90.72% sensitivity, 96.76% specificity
Kim et al. 2024	AD severity classification	ResNet-50 with HSI	83% classification accuracy
Sulejmani et al. 2024	Patient query response	Large language model	Outperformed GPs in quality and empathy

## Role of AI in the treatment of AD

### Formulation of personalized treatment plans

AI is gradually becoming a vital tool in the evolving landscape of personalized health. By considering patients’ genetic makeup, lifestyle, environment, and clinical signs, AI can tailor treatment regimens to suit individual patients more effectively [39]. The traditional one-size-fits-all approach to managing skin diseases is inadequate and need be replaced. By applying deep learning to patients’ genetic history, skin microbiome characteristics, medical history, and observable symptoms, AI accurately predicts factors that might influence disease severity and treatment response [40,41].

Applying AI in treating AD has shown remarkable success, mainly through advanced AI technologies in developing treatment plans. Models such as random forest, SVM, and CNN analyze extensive datasets, including gene expression data and clinical history, to identify response patterns across various patient sub-populations [42]. This approach ensures efficient and tailored treatment strategies, increasing positive outcomes and reducing the adverse effects of ineffective treatments. Furthermore, AI integrates diverse datasets to provide comprehensive information, further supporting the development of individualized treatment plans. An exploratory analysis based on the Japanese Environmental and Child Study, using machine learning techniques to study plasma cytokine/chemokine levels in 6-year-old children, found that atopic dermatitis (AD) is associated with specific chemokines CCL17 and Association with elevated CCL27 levels. In addition, using AI technology, the study divided children with asthma symptoms into two different biomarker groups, revealing potential avenues for personalized treatment [43].

### Application in AD drug development

Integrating AI technology into drug discovery and development transforms the conventional approach by reducing costs and timeframes associated with bringing new drugs to market. In drug development for treating AD, AI plays a crucial role in enhancing the process and optimizing the administration of clinical trials.

#### Optimization of drug development process

During the initial stages of drug discovery, AI accelerates the screening process by using computer models to simulate interactions between compounds and biological targets [44]. Employing ML and deep-learning algorithms, AI screens through databases containing millions of compounds, predicting their biological activities and potential side effects. This approach significantly expedites drug discovery timelines while minimizing the research and development expenditures [45]. For instance, A research team led by Professor Yoshihiro Yamanishi developed an innovative computational method, named DRAGONET, focusing on the treatment of AD. This method uses deep learning technology to automatically generate new drug molecular structures with therapeutic potential based on patients’ gene expression data [46]. The research team first identified Disease Reversal Molecules (DRMs) that were inversely related to specific gene expression patterns of atopic dermatitis, and then used the variational autoencoder (VAE) model, especially the transformer-based VAE model to explore the latent space and generate new molecular structures. Specifically, the 1st DRM identified in the study contains a variety of nitrogen atoms and ring structures, while the newly generated molecules have undergone structural optimization while maintaining the six-membered ring structure, such as transforming a benzene ring into a pyridine ring, which is closer to the structure of pyridoxal phosphate hydrate, a registered drug for the treatment of atopic dermatitis. They applied DRAGONET to generate drug candidate molecules for atopic dermatitis, and demonstrated that the newly generated molecules were chemically similar to registered drugs for each disease.

#### Multi-target drug screening and optimization

Through the integration of network analysis, deep learning, and molecular simulation techniques, the research team identified caffeoyl malic acid (CMA) from Taiwan’s traditional Chinese medicine database as a promising dual inhibitor of TNFα/IL-4 for atopic dermatitis. Molecular simulations demonstrated that CMA exhibits strong binding affinity to TNF-α and IL-4, potentially altering protein conformation and impeding the formation of protein-receptor complexes [47]. These findings suggest the potential of CMA as a therapeutic agent for atopic dermatitis. AI has revolutionized the drug discovery process for treating AD by enhancing the understanding interactions between compounds and biological targets. AI enables rapid virtual screening of extensive compound libraries, identifies drug molecules with multi-target activities, and predicts their biological effectiveness and potential toxicity. Such measures accelerate drug discovery and improve success rates compared to traditional methods.

#### Optimization of clinical trials

Artificial intelligence technology plays a critical role in drug clinical trials, particularly in patient recruitment and data analysis phases. By leveraging electronic health records, AI efficiently sifts through vast amounts of medical data to pinpoint eligible patients, saving time and reducing errors. This approach is frequently utilized for patient enrollment in studies related to melanoma, psoriasis, and different types of tumors [48]. Moreover, artificial intelligence monitors and assesses real-time test data to identify anomalies and trends, thereby enhancing the credibility and efficiency of trials. It aids in the identification of side effects and prediction of treatment success by analyzing patient response data [49].

### Predicting treatment response and monitoring therapeutic effects

Among those with AD, capturing patient responses to specific management approaches and monitoring therapeutic efficacy in real time is essential. As AI progresses, it will enable a better understanding of patient reactions to different treatments and facilitate real-time management of treatment effectiveness, allowing adjustments as necessary.

#### Predicting patient responses to different treatment regimens

Patient characteristics significantly influence treatment outcomes in AD [50]. AI technology can predict patient’s sensitivity and potential responses to specific treatment plans based on genetics, skin lesion data, and prior treatment records. A study conducted by the Dermatology Research and Education Foundation and Pfizer utilized machine learning to analyze data from 419 atopic dermatitis patients in order to predict their response to dupilumab treatment. The findings revealed that 35% of patients exhibited signs of non-response to the treatment, with treatment discontinuation being the most prevalent indicator. Notable predictive factors identified in the study included patient usage of ibuprofen and a higher Quan-Charlson comorbidity index [51]. These research findings contribute to the advancement of clinical prediction for atopic dermatitis and offer insights to enhance disease management strategies.

Moreover, an innovative study extended the application of AI in medical diagnostics by introducing the bSRWPSO-FKNN intelligent model. The study conducted 10 rounds of 10-fold cross-validation experiments on a medical dataset with 181 atopic dermatitis patients to validate the predictive capability of the bSRWPSO-FKNN model. Results indicate that the model successfully identified key features influencing the onset of atopic dermatitis, such as lymphocyte content (LY), cat dander, milk, and total IgE. The combination of these features, along with nine public and medical datasets, demonstrates the scientific rigor and practical value of the bSRWPSO-FKNN model in predicting AD [52]. The potential of AI in aiding the prediction of atopic dermatitis treatment has been recognized for its long-term development.

#### Real-time monitoring and adjustment of therapeutic effects

Real-time monitoring and AI-aided diagnosis are vital for optimizing patient care. Aaron M. Smith ‘s study, utilizing deep learning for disease prediction in conditions such as UC, AD, and cancers, demonstrates the impact of gene selection, standardization, and algorithms on accuracy. Their work highlights the potential of deep learning with proper preprocessing and algorithm choice for enhancing diagnostic accuracy. This approach aids in developing AI tools for analyzing large datasets, supporting real-time treatment monitoring. The study sets benchmarks for AI prediction models and provides resources for exploring large-scale datasets like GTEx and TCGA, emphasizing the need for ML to handle complex data for improving model generalizability and accuracy [53].

**Figure 2.**
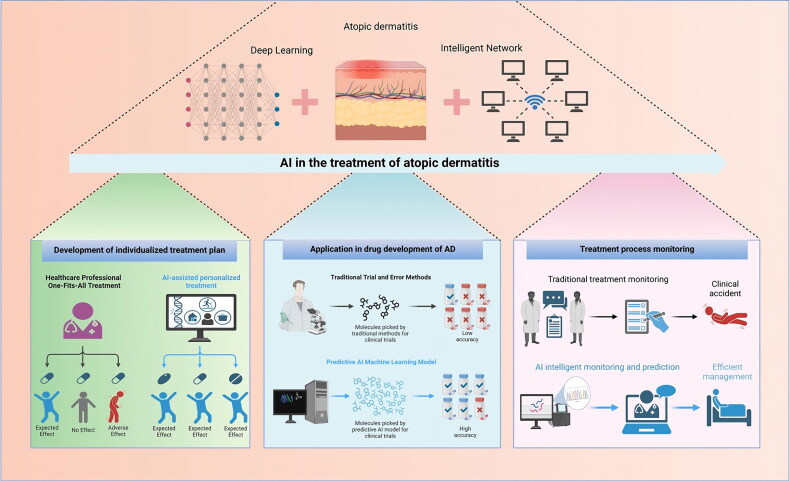
The role of artificial intelligence in atopic dermatitis treatment.

Artificial intelligence can assist patients with atopic dermatitis in achieving improved therapeutic outcomes through the management of personalized treatment, drug development, prediction, optimization of clinical trials, and efficacy forecasting (Figure 2).

## AI applications in AD patient management

### Self-management and disease monitoring

Self-management plays a crucial role in sustaining remission among patients with AD. AI-powered self-monitoring applications empower patients to track changes in their condition, medication adherence, and behavioral patterns in real-time using smart devices and applications. These devices gather daily data on skin condition, itchiness, humidity, temperature, and other parameters, which are then transmitted to the cloud for analysis. The research team, led by Martin Kraepelien, has developed a digital self-service intervention tool for atopic dermatitis (AD). This tool includes exposure and mindfulness exercises to track symptoms and triggers through a digital diary, significantly reducing participant workload. In cross-validation experiments, the within-group effect size of the new tool at the 3-month follow-up (Cohen’s *d* = 0.84) was found to be similar to that of a comprehensively guided intervention, suggesting the potential of digital psychological self-help tools in treating atopic dermatitis [54]. The study findings indicated that patients who utilized the tool exhibited improved self-management skills and a decrease in the frequency of exacerbations. A study with 21 Swedish AD patients demonstrated that a self-help intervention utilizing cognitive behavioral therapy (CBT) helped patients access health information and enhance their understanding of the condition [55]. This intervention notably enhanced patients’ quality of life, decreased itching, and reduced levels of depression and stress, with effects ranging from moderate to significant.

Overall, AI tools expand on previous web-based interventions supervised by clinicians, saving clinician time by simplifying and optimizing the user interface, and reducing participant reading while maintaining group engagement comparable to fully supervised web-based interventions in terms of within effect sizes and response rates.

### Health education and psychological support

Educational support and psychological care play a crucial role in the successful management of AD [56]. The integration of artificial intelligence technology has facilitated the creation of personalized health education platforms that offer customized health information and educational resources. For instance, the recently introduced mobile health application AtopipApp delivers educational content on AD epidemiology, severity progression, and treatment options. This app is specifically tailored to assist parents in managing AD in their children. The significant reduction in the POEM score among users of the Atopic App indicates a potential impact of this tool on health care engagement by caregivers of children with AD. These applications empower patients to access disease management resources conveniently, acquire practical nursing skills, and enhance their understanding and management capabilities of the condition [57].

Furthermore, AI technology enables real-time psychological support through virtual health assistants. These assistants interact with patients, addressing their inquiries about disease management and providing emotional support. This continuous psychological support effectively alleviates patients’ anxiety and depression, improving their overall mental health. By offering personalized health education and psychological support, AI technology plays a significant role in the long-term care of patients with AD. It helps them effectively cope with the psychological challenges associated with the disease, enhancing their quality of life.

### AI applications in telemedicine

Telemedicine is becoming increasingly important in managing AD, especially in remote areas or during unique circumstances. AI technology enhances telemedicine services by making them more efficient and accessible [58]. Two studies, one by Karolinska Institute in Sweden and the other by Kevin Zarca’s team in a French prison, showcased the use of AI telemedicine in treating atopic dermatitis (AD). Kern’s study focused on a digital self-help intervention based on cognitive behavioral therapy (CBT), which showed moderate to large improvements in 21 adult AD patients over 8 weeks. Zarca’s research, on the other hand, demonstrated a significant increase in treatment plan completion rates among inmate patients through a remote expert network. Both studies underscore the benefits of AI telemedicine in offering convenient, cost-effective, and efficacious AD treatment across various settings, including unique environments like prisons [55,59].

**Figure 3.**
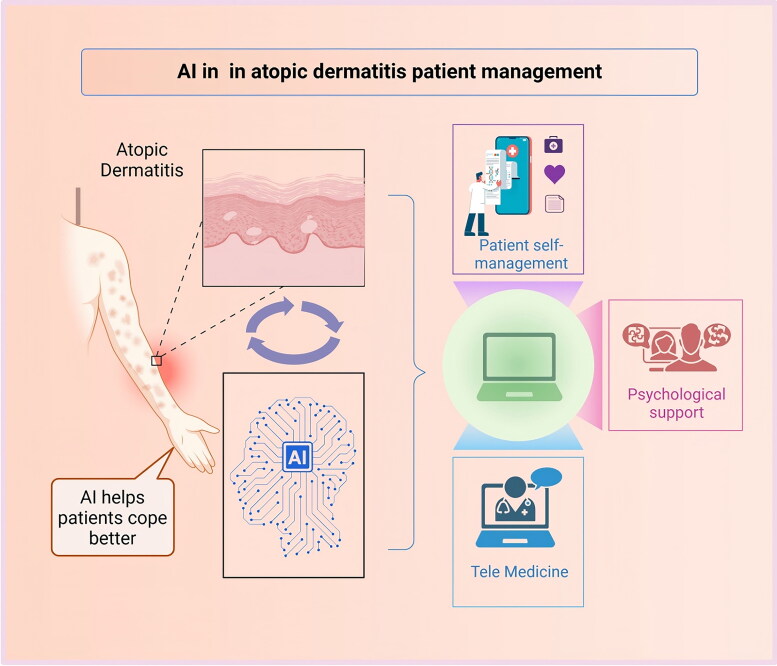
Application of artificial intelligence in the management of atopic dermatitis patients.

In addition, artificial intelligence technology has significantly improved the efficiency of remote monitoring and follow-up of dermatological patients. Currently, artificial intelligence remote detection systems have been developed for diseases such as psoriasis, melanoma and skin burns. For example, the image-based YOLO-v4 algorithm performs remote assessment of psoriasis; Australian health services use artificial intelligence systems to remotely assess skin wounds; and a team uses computer deep learning and artificial intelligence to provide remote guidance to patients with skin burns [60–62]. This series of AI applications can help with clinical judgment or treatment planning. With AI-driven decision support systems integrated into remote monitoring platforms, healthcare providers can make more informed clinical decisions and optimize resource allocation. Allows doctors to monitor changes in a patient’s condition in real time and adjust treatment plans if necessary (Figure 3 and Table 2).

**Table 2.** Current AI-based tools and applications for AD management.

Tool name	Type	Main functions	Main functions & algorithms
Diagnostic tools			
ResNet-50 with HSl	Image analysis	AD severity classification and diagnosis	Residual neural network
VGGNet-19	Image analysis	AD severity assessment	Deep convolutional neural network
MobileNetV3	Image analysis	AD severity assessment	Lightweight CNN for mobile devices
MnasNet	Image analysis	AD severity assessment	Mobile neural architecture search
EfficientNetB0	Image analysis	AD severity assessment	Compound scaling optimization
Multiphoton tomography with CNN	Advanced imaging	Cell identification and AD diagnosis	CNN with deep Taylor decomposition
PLS-DA model with CRM	Biomarker analysis	Gene expression profile analysis	Partial least squares discriminant
Treatment tools			
DRAGONET	Drug development	Novel drug molecule generation	Variational autoencoder (VAE)
MedlA	Data management	Patient data integration and analysis	Multimodal algorithms
ML-based Dupilumab response predictor	Treatment response	Treatment response prediction	Supervised machine learning
bSRWPSO-FKNN	Disease prediction	AD onset prediction	Boosted PSO with fuzzy KNN classifier
Patient management tools			
AtopipApp	Mobile health	Education and disease monitoring	Personalized content algorithms
Digital CBT self-help tool	Psychological support	Self-management intervention	CBT algorithms with AI personalization
YOLO-v4 algorithm	Remote monitoring	Remote lesion assessment	Real-time object detection
Al virtual health assistant	Patient support	Real-time patient guidance	NLP and machine learning

## Challenges and prospects

### Opportunities and challenges in AI application

Advancements in artificial intelligence (AI) for atopic dermatitis (AD) diagnosis and treatment show promise but are not without hurdles. The efficacy of AI is contingent on accessing comprehensive and varied medical data. However, this is impeded by privacy concerns, data standardization challenges, and institutional interoperability gaps. Moreover, the ‘black box’ nature of AI models, which are often opaque, can erode trust among healthcare professionals and patients, emphasizing the need for transparency to ensure AI-assisted decisions are embraced and effective.

Despite these challenges, AI’s potential in revolutionizing AD management is vast. It offers the ability to predict diseases, facilitate early diagnoses, and craft personalized risk assessments and preventative strategies that consider individual genetic, lifestyle, and environmental factors. AI also accelerates drug discovery, offering patients a wider array of treatment options. The integration of AI in telemedicine and mHealth is set to enhance disease management by providing real-time monitoring and personalized care, thereby improving life quality for patients. To harness AI’s full potential, there is a pressing need for robust data infrastructure, transparent algorithms, and robust ethical and policy frameworks. Interdisciplinary cooperation is key to standardizing data practices, ensuring algorithmic fairness, and safely and responsibly integrating AI into AD management.

### The expanding horizons of AI in atopic dermatitis treatment

With the advancement of artificial intelligence technology, its utilization in the management of atopic dermatitis is poised to grow and enhance. Beyond just image recognition and data analysis, artificial intelligence is at the brink of transforming the complete disease management process. From detecting the disease early on to continuously monitoring and tailoring treatment plans, AI’s profound learning abilities will allow it to forecast disease progression and offer personalized risk evaluations and preventive actions by analyzing patient-specific data from multiple dimensions. This predictive power significantly enhances early diagnosis accuracy, enabling preemptive intervention prior to symptom manifestation. Throughout the treatment process, AI will continuously adjust treatment options by analyzing patients’ physiological responses and treatment outcomes in real-time. This approach promotes precision medicine and reduces the occurrence of adverse drug reactions.

In the field of drug discovery, artificial intelligence is anticipated to expedite the development and approval of new drugs, expanding the options for patients to access advanced and efficient treatments. When combined with telemedicine and mobile health technologies, AI will enhance the accessibility of expert medical monitoring and consultations, making top-notch healthcare more readily available and convenient. This integrated strategy for managing diseases aims to empower patients by equipping them with the necessary tools to effectively oversee their health and enhance their overall well-being.

## Conclusion

In summary, our study explores the innovative applications of artificial intelligence (AI) technology in the diagnosis, treatment, and management of atopic dermatitis (AD), highlighting the significant value of AI technology in enhancing diagnostic accuracy, customizing personalized treatment plans, streamlining drug development processes, and facilitating remote medical care. AI technology leverages deep learning, big data analysis, and pattern recognition to notably improve skin lesion recognition, enabling more precise and individualized treatment plans for patients. Furthermore, it optimizes the design and oversight of clinical trials. Despite challenges such as data privacy concerns, algorithm transparency issues, and ethical considerations; the prospects for AI application in AD management are promising with an expected pivotal role in shaping future advancements in skin health care delivery that will ultimately lead to improved medical services quality and enhanced patient quality of life.

## Data Availability

Data sharing not applicable to this article as no datasets were generated or analysed during the current study.

## References

[1] Biazus Soares G, Hashimoto T, Yosipovitch G. Atopic dermatitis itch: scratching for an explanation. J Invest Dermatol. 2024;144(5):978–988. doi: 10.1016/j.jid.2023.10.048. 38363270

[2] Choo ZY, Mehlis SL, Joyce JC. Updates in atopic dermatitis for the primary care physician: a review of advances in the understanding and treatment of atopic dermatitis. Dis Mon. 2024;70(4):101687. doi: 10.1016/j.disamonth.2024.101687. 38278753

[3] Faye O, Flohr C, Kabashima K, et al. Atopic dermatitis: a global health perspective. J Eur Acad Dermatol Venereol. 2024;38(5):801–811. doi: 10.1111/jdv.19723. 38151270

[4] Nazarian A, Alexis AF. Diagnosis of allergic dermatoses in skin of color. Curr Allergy Asthma Rep. 2024;24(6):317–322. doi: 10.1007/s11882-024-01148-8. 38776041

[5] Afshari M, Kolackova M, Rosecka M, et al. Unraveling the skin: a comprehensive review of atopic dermatitis, current understanding, and approaches. Front Immunol. 2024;15:1361005. doi: 10.3389/fimmu.2024.1361005. 38500882 PMC10944924

[6] Trogen B, Jacobs S, Wang J. Disparities in the diagnosis and management of anaphylaxis. Curr Allergy Asthma Rep. 2023;23(1):13–19. doi: 10.1007/s11882-022-01053-y. 36454450

[7] Armario-Hita JC, Galán-Gutiérrez M, Dodero-Anillo JM, et al. Updated review on treatment of atopic dermatitis. J Investig Allergol Clin Immunol. 2023;33(3):158–167. doi: 10.18176/jiaci.0906. 37318771

[8] Hagenström K, Klinger T, Müller K, et al. Utilization and related harms of systemic glucocorticosteroids for atopic dermatitis: claims data analysis. Br J Dermatol. 2024;191(5):719–727. doi: 10.1093/bjd/ljae250. 38924726

[9] Ali F, Vyas J, Finlay AY. Counting the burden: atopic dermatitis and health-related quality of life. Acta Derm Venereol. 2020;100(12):adv00161. doi: 10.2340/00015555-3511. 32412644 PMC9189752

[10] Davis DM, Waldman A, Jacob S, et al. Diagnosis, comorbidity, and psychosocial impact of atopic dermatitis. Semin Cutan Med Surg. 2017;36(3):95–99. doi: 10.12788/j.sder.2017.028. 28895954

[11] Hogarty DT, Su JC, Phan K, et al. Artificial intelligence in dermatology-where we are and the way to the future: a review. Am J Clin Dermatol. 2020;21(1):41–47. doi: 10.1007/s40257-019-00462-6. 31278649

[12] Young AT, Xiong M, Pfau J, et al. Artificial intelligence in dermatology: a primer. J Invest Dermatol. 2020;140(8):1504–1512. doi: 10.1016/j.jid.2020.02.026. 32229141

[13] Mehta PP, Sun M, Betz-Stablein B, et al. Improving artificial intelligence-based diagnosis on pediatric skin lesions. J Invest Dermatol. 2023;143(8):1423–1429.e1. doi: 10.1016/j.jid.2022.08.058. 36804150 PMC10431965

[14] Sufyan M, Shokat Z, Ashfaq UA. Artificial intelligence in cancer diagnosis and therapy: current status and future perspective. Comput Biol Med. 2023;165:107356. doi: 10.1016/j.compbiomed.2023.107356. 37688994

[15] Du-Harpur X, Watt FM, Luscombe NM, et al. What is AI? Applications of artificial intelligence to dermatology. Br J Dermatol. 2020;183(3):423–430. doi: 10.1111/bjd.18880. 31960407 PMC7497072

[16] van der Velden BHM, Kuijf HJ, Gilhuijs KGA, et al. Explainable artificial intelligence (XAI) in deep learning-based medical image analysis. Med Image Anal. 2022;79:102470. doi: 10.1016/j.media.2022.102470. 35576821

[17] Kim EB, Baek YS, Lee O. Parameter-based transfer learning for severity classification of atopic dermatitis using hyperspectral imaging. Skin Res Technol. 2024;30(4):e13704. doi: 10.1111/srt.13704. 38627927 PMC11021799

[18] Maulana A, Noviandy TR, Suhendra R, et al. Evaluation of atopic dermatitis severity using artificial intelligence. Narra J. 2023;3(3):e511. doi: 10.52225/narra.v3i3.511. 38450339 PMC10914065

[19] Bang CH, Yoon JW, Ryu JY, et al. Automated severity scoring of atopic dermatitis patients by a deep neural network. Sci Rep. 2021;11(1):6049. doi: 10.1038/s41598-021-85489-8. 33723375 PMC7961024

[20] Medela A, Mac Carthy T, Aguilar Robles SA, et al. automatic scoring of atopic dermatitis using deep learning: a pilot study. JID Innov. 2022;2(3):100107. doi: 10.1016/j.xjidi.2022.100107. 35990535 PMC9382656

[21] Park S, Saw SN, Li X, et al. Model learning analysis of 3D optoacoustic mesoscopy images for the classification of atopic dermatitis. Biomed Opt Express. 2021;12(6):3671–3683. doi: 10.1364/BOE.415105. 34221687 PMC8221944

[22] Burns BL, Rhoads DD, Misra A. The use of machine learning for image analysis artificial intelligence in clinical microbiology. J Clin Microbiol. 2023;61(9):e0233621. doi: 10.1128/jcm.02336-21. 37395657 PMC10575257

[23] Dev K, Ho CJH, Bi R, et al. Machine learning assisted handheld confocal Raman micro-spectroscopy for identification of clinically relevant atopic eczema biomarkers. Sensors (Basel). 2022;22(13):4674. doi: 10.3390/s22134674. 35808168 PMC9269422

[24] Jiang Z, Li J, Kong N, et al. Accurate diagnosis of atopic dermatitis by combining transcriptome and microbiota data with supervised machine learning. Sci Rep. 2022;12(1):290. doi: 10.1038/s41598-021-04373-7. 34997172 PMC8741793

[25] Zhou B, Zhou N, Liu Y, et al. Identification and validation of CCR5 linking keloid with atopic dermatitis through comprehensive bioinformatics analysis and machine learning. Front Immunol. 2024;15:1309992. PMID: 38476235; PMCID: PMC10927814. doi: 10.3389/fimmu.2024.1309992. 38476235 PMC10927814

[26] Wu W, Chen G, Zhang Z, et al. Construction and verification of atopic dermatitis diagnostic model based on pyroptosis related biological markers using machine learning methods. BMC Med Genom. 2023;16(1):138. PMID: 37330465; PMCID: PMC10276470. doi: 10.1186/s12920-023-01552-5. PMC10276470 37330465

[27] Miller DD, Brown EW. Artificial intelligence in medical practice: the question to the answer? Am J Med. 2018;131(2):129–133. doi: 10.1016/j.amjmed.2017.10.035. 29126825

[28] Sulejmani P, Negris O, Aoki V, et al. A large language model artificial intelligence for patient queries in atopic dermatitis. J Eur Acad Dermatol Venereol. 2024;38(6):e531–e5. doi: 10.1111/jdv.19737. 38168874

[29] Abdalla M, Chen B, Santiago R, et al. Accuracy of algorithms to identify people with atopic dermatitis in ontario routinely collected health databases. J Invest Dermatol. 2021;141(7):1840–1843. doi: 10.1016/j.jid.2021.01.009. 33571528

[30] Acosta JN, Falcone GJ, Rajpurkar P, et al. Multimodal biomedical AI. Nat Med. 2022;28(9):1773–1784. doi: 10.1038/s41591-022-01981-2. 36109635

[31] Luo N, Zhong X, Su L, et al. Artificial intelligence-assisted dermatology diagnosis: from unimodal to multimodal. Comput Biol Med. 2023;165:107413. doi: 10.1016/j.compbiomed.2023.107413. 37703714

[32] Ohta T, Hananoe A, Fukushima-Nomura A, et al. Best practices for multimodal clinical data management and integration: an atopic dermatitis research case. Allergol Int. 2024;73(2):255–263. doi: 10.1016/j.alit.2023.11.006. 38102028

[33] Guimarães P, Batista A, Zieger M, et al. Artificial intelligence in multiphoton tomography: atopic dermatitis diagnosis. Sci Rep. 2020;10(1):7968. doi: 10.1038/s41598-020-64937-x. 32409755 PMC7224284

[34] Mohsen F, Ali H, El Hajj N, et al. Artificial intelligence-based methods for fusion of electronic health records and imaging data. Sci Rep. 2022;12(1):17981. doi: 10.1038/s41598-022-22514-4. 36289266 PMC9605975

[35] Liang H, Tsui BY, Ni H, et al. Evaluation and accurate diagnoses of pediatric diseases using artificial intelligence. Nat Med. 2019;25(3):433–438. doi: 10.1038/s41591-018-0335-9. 30742121

[36] Felmingham C, Pan Y, Kok Y, et al. Improving skin cancer management with artificial intelligence: a pre-post intervention trial of an artificial intelligence system used as a diagnostic aid for skin cancer management in a real-world specialist dermatology setting. J Am Acad Dermatol. 2023;88(5):1138–1142. doi: 10.1016/j.jaad.2022.10.038. 36306873

[37] Huang HY, Nguyen HT, Lin TL, et al. Identification of skin lesions by snapshot hyperspectral imaging. Cancers (Basel). 2024;16(1):217. doi: 10.3390/cancers16010217. 38201644 PMC10778186

[38] Zaar O, Larson A, Polesie S, et al. Evaluation of the diagnostic accuracy of an online artificial intelligence application for skin disease diagnosis. Acta Derm Venereol. 2020;100(16):adv00260. doi: 10.2340/00015555-3624. 32852557 PMC9234984

[39] Subramanian M, Wojtusciszyn A, Favre L, et al. Precision medicine in the era of artificial intelligence: implications in chronic disease management. J Transl Med. 2020;18(1):472. doi: 10.1186/s12967-020-02658-5. 33298113 PMC7725219

[40] Chen ZH, Lin L, Wu CF, et al. Artificial intelligence for assisting cancer diagnosis and treatment in the era of precision medicine. Cancer Commun (Lond). 2021;41(11):1100–1115. doi: 10.1002/cac2.12215. 34613667 PMC8626610

[41] Belge Bilgin G, Bilgin C, Burkett BJ, et al. Theranostics and artificial intelligence: new frontiers in personalized medicine. Theranostics. 2024;14(6):2367–2378. doi: 10.7150/thno.94788. 38646652 PMC11024845

[42] Maintz L, Welchowski T, Herrmann N, et al. Machine learning-based deep phenotyping of atopic dermatitis: severity-associated factors in adolescent and adult patients. JAMA Dermatol. 2021;157(12):1414–1424. doi: 10.1001/jamadermatol.2021.3668. 34757407 PMC8581798

[43] Yamamoto-Hanada K, Kawakami E, Saito-Abe M, et al. Exploratory analysis of plasma cytokine/chemokine levels in 6-year-old children from a birth cohort study. Cytokine. 2020;130:155051. doi: 10.1016/j.cyto.2020.155051. 32151964

[44] Mak KK, Pichika MR. Artificial intelligence in drug development: present status and future prospects. Drug Discov Today. 2019;24(3):773–780. doi: 10.1016/j.drudis.2018.11.014. 30472429

[45] Gupta R, Srivastava D, Sahu M, et al. Artificial intelligence to deep learning: machine intelligence approach for drug discovery. Mol Divers. 2021;25(3):1315–1360. doi: 10.1007/s11030-021-10217-3. 33844136 PMC8040371

[46] Yamanaka C, Uki S, Kaitoh K, et al. De novo drug design based on patient gene expression profiles via deep learning. Mol Inform. 2023;42(8–9):e2300064. doi: 10.1002/minf.202300064. 37475603

[47] Wang Y, Qin D, Jin L, et al. Caffeoyl malic acid is a potential dual inhibitor targeting TNFα/IL-4 evaluated by a combination strategy of network analysis-deep learning-molecular simulation. Comput Biol Med. 2022;145:105410. doi: 10.1016/j.compbiomed.2022.105410. 35325732

[48] Harrer S, Shah P, Antony B, et al. Artificial intelligence for clinical trial design. Trends Pharmacol Sci. 2019;40(8):577–591. doi: 10.1016/j.tips.2019.05.005. 31326235

[49] Lin Y, Zhang Y, Wang D, et al. Computer especially AI-assisted drug virtual screening and design in traditional Chinese medicine. Phytomedicine. 2022;107:154481. doi: 10.1016/j.phymed.2022.154481. 36215788

[50] Huet F, Faffa MS, Poizeau F, et al. Characteristics of pruritus in relation to self-assessed severity of atopic dermatitis. Acta Derm Venereol. 2019;99(3):279–283. doi: 10.2340/00015555-3053. 30264161

[51] Wu JJ, Hong CH, Merola JF, et al. Predictors of nonresponse to dupilumab in patients with atopic dermatitis: a machine learning analysis. Ann Allergy Asthma Immunol. 2022;129(3):354–359.e5. doi: 10.1016/j.anai.2022.05.025. 35640774

[52] Li Y, Zhao D, Xu Z, et al. bSRWPSO-FKNN: a boosted PSO with fuzzy K-nearest neighbor classifier for predicting atopic dermatitis disease. Front Neuroinform. 2022;16:1063048. doi: 10.3389/fninf.2022.1063048. 36726405 PMC9884708

[53] Smith AM, Walsh JR, Long J, et al. Standard machine learning approaches outperform deep representation learning on phenotype prediction from transcriptomics data. BMC Bioinformat. 2020;21(1):119. doi: 10.1186/s12859-020-3427-8. PMC7085143 32197580

[54] Kraepelien M, Hentati A, Kern D, et al. Transforming guided internet interventions into simplified and self-guided digital tools – experiences from three recent projects. Internet Interv. 2023;34:100693. doi: 10.1016/j.invent.2023.100693. 38073676 PMC10709100

[55] Kern D, Ljótsson B, Lönndahl L, et al. A digital self-help intervention for atopic dermatitis: analysis of secondary outcomes from a feasibility study. JMIR Dermatol. 2023;6:e42360. doi: 10.2196/42360. 37632924 PMC10335136

[56] Fasseeh AN, Elezbawy B, Korra N, et al. Burden of atopic dermatitis in adults and adolescents: a systematic literature review. Dermatol Ther (Heidelb). 2022;12(12):2653–2668. doi: 10.1007/s13555-022-00819-6. 36197589 PMC9674816

[57] Zvulunov A, Lenevich S, Migacheva N. A mobile health app for facilitating disease management in children with atopic dermatitis: feasibility and impact study. JMIR Dermatol. 2023;6:e49278. doi: 10.2196/49278. 38090787 PMC10753416

[58] Tiribelli S, Monnot A, Shah SFH, et al. Ethics principles for artificial intelligence-based telemedicine for public health. Am J Public Health. 2023;113(5):577–584. doi: 10.2105/AJPH.2023.307225. 36893365 PMC10088937

[59] Zarca K, Charrier N, Mahé E, et al. Tele-expertise for diagnosis of skin lesions is cost-effective in a prison setting: a retrospective cohort study of 450 patients. PLoS One. 2018;13(9):e0204545. doi: 10.1371/journal.pone.0204545. 30248151 PMC6152874

[60] Ethier O, Chan HO, Abdolahnejad M, et al. Using computer vision and artificial intelligence to track the healing of severe burns. J Burn Care Res. 2024;45(3):700–708. doi: 10.1093/jbcr/irad197. 38126807

[61] Yin H, Chen H, Zhang W, et al. Image-based remote evaluation of PASI scores with psoriasis by the YOLO-v4 algorithm. Exp Dermatol. 2024;33(4):e15082. doi: 10.1111/exd.15082. 38664884

[62] Barakat-Johnson M, Jones A, Burger M, et al. Reshaping wound care: evaluation of an artificial intelligence app to improve wound assessment and management. Stud Health Technol Inform. 2024;310:941–945. doi: 10.3233/SHTI231103. 38269947

